# Early life experience and alterations of group composition shape the social grooming networks of former pet and entertainment chimpanzees *(Pan troglodytes)*

**DOI:** 10.1371/journal.pone.0226947

**Published:** 2020-01-15

**Authors:** Dietmar Crailsheim, Hans Peter Stüger, Elfriede Kalcher-Sommersguter, Miquel Llorente

**Affiliations:** 1 Unitat de Recerca i Etologia, Fundació MONA, Riudellots de la Selva, Spain; 2 Facultat d’Educació i Psicologia, Universitat de Girona, Girona, Spain; 3 Department of Statistics and Analytical Epidemiology, AGES - Austrian Agency for Health and Food Safety, Graz, Austria; 4 Institute of Biology, University of Graz, Graz, Austria; 5 IPRIM - Institut de Recerca i Estudis en Primatologia, Girona, Spain; Istituto Superiore Di Sanita, ITALY

## Abstract

The long-term effects of early life adversities on social capacities have been documented in humans and wild-caught former laboratory chimpanzees *(Pan troglodytes)*. However, former pet and entertainment chimpanzees have received little attention to date. This study aimed to investigate the long-term effects of early life experience on 18 former pet and entertainment chimpanzees, based on social grooming data collected at a primate rescue centre over a 12-year period. Moreover, we also focused on the possible short-term effects that alterations to group composition might have on grooming patterns. For this purpose, we compared stable and unstable periods (i.e. where alterations to group composition occurred). We used two individual social network measures to analyse the grooming activity and the distribution of grooming among group mates for each individual. We could show that wild-caught chimpanzees were significantly more selective regarding their grooming partners and spent less time grooming when compared to their captive born companions. We also found that individuals who were predominantly housed without conspecifics during infancy spent less time grooming compared to those who were predominantly housed with conspecifics during infancy. Furthermore, we found that alterations to the group composition had short-term effects on the distribution of social grooming from a more equal distribution during periods with a stable group composition towards a more unequal and selective distribution during unstable periods. Thus, we conclude that the social grooming networks of former pet and entertainment chimpanzees are shaped not only by long-term effects such as early life experience, but also by short-term effects such as alterations to group composition. Remarkably, we found not only captive born chimpanzees but also wild-caught individuals to adjust their grooming to socially challenging situations by modifying their grooming distribution in a similar way.

## Introduction

Adverse experiences in early infancy affect the behaviour as well as the physical and mental health of human [1–3] and non-human primates [4, 5] in the long term [6–9]. This applies to individual as well as social behaviour [10]. Wild infant chimpanzees spend their first two to five years of life either attached to or in close proximity to their mothers [11–13], and develop their social skills by interacting with their mother and other members of their group [12, 14]. The developmental trajectory of chimpanzees is similar to that of humans [12], and their cognitive [15, 16], emotional [17] and social skills [12] are highly complex. This complexity is reflected in their social organization as well. Chimpanzees in the wild use a fission-fusion system, which implies regular transfers of individuals between small subgroups, increasing the individuals’ survival chances and group functionality [18–20]. Between the 1950s and the 1980s, thousands of infant chimpanzees were taken from the wild [21, 22]. These orphans experienced the separation from their mother and most likely witnessed the killing of their mother and other group members [23, 24]. Furthermore, they experienced a dramatic change in living conditions from wild to captivity and were transported to the United States of America, Asia and Europe under deplorable conditions [25, 26]. Many of the imported orphans were used for biomedical research, but were also used for entertainment or kept as pets for decades. These orphans experienced early maternal loss [26, 27], and often additionally to this, prolonged solitary housing [28] and the lack of diverse conspecific social partners [29, 30]. Growing up as an orphan can have deleterious effects later in life in humans [31], chimpanzees [32] and other non-human primates [33]. Several studies suggest that early life stress induces long-term morphologic changes in primate brains expressing a delayed effect once the vulnerable brain system reaches maturation [34, 35].

While there are studies focusing on the lasting effects of laboratory housing on nonhuman primates [36, 37], less is known in this regard about former pet (i.e. privately owned and kept for companionship or pleasure) and entertainment (i.e. were trained and used for commercial purpose) chimpanzees. Some studies report that extensive human exposure during the first years of life had an effect on the behaviour of former pet and entertainment chimpanzees. For example, Freeman and Ross [4] found that chimpanzees, living in accredited zoos and sanctuaries, who had more exposure to humans as infants exhibited less social grooming and sexual behaviours than chimpanzees with more conspecific exposure during infancy. Likewise, Llorente and colleagues [10] found that chimpanzees living in a sanctuary who were younger at the onset of rehabilitation spent more time with social play and affiliative behaviours, less time inactive, and reached higher levels of behavioural and social competence than individuals who started their rehabilitation at an older age. Furthermore, former pet and entertainment chimpanzees, living in accredited zoos and sanctuaries, who were mainly exposed to humans in infancy showed lower levels of extraversion and exhibited deficiencies in social bonding [38]. Finally, Ortín et al. [39] demonstrated that the personality development of chimpanzees who were rescued from bushmeat and pet trade was affected by these early life experiences, and resulted in higher ratings in the factors anxiety, restraint and dominance.

Social network analysis (SNA) allows us to statistically describe, quantify and compare the social relationships of individuals within a group [40–42]. Although it has been proven to be an extremely useful tool for describing complex social systems and investigating welfare of animals, few studies have focused on former pet and performer chimpanzees in zoos and rescue centres to date [43, 44]. By detecting social patterns on a group level and analysing asymmetries of certain individuals under specific conditions, we can go further and identify factors influencing social interactions and social group structures. As such, SNA can also be used as an animal welfare tool and can play a supporting role in animal management decisions [42, 45]. Investigating the social networks of former pet and entertainment chimpanzees has the potential to provide a better understanding of the long-term implications of early life adversities and to improve animal welfare [42, 46].

The recovery from early life adversities is a long lasting process, but can, at least in part, be achieved through social, psychological, emotional and environmental interventions in specialized institutions like primate rescue and rehabilitation centres [10, 47]. These centres strive to recreate living conditions similar to that of wild living conspecifics, i.e. permanent access to other conspecifics and occasional changes of group composition [48]. However, while chimpanzees living in the wild decide autonomously to move between subgroups, the social setting and any changes to the group composition are handled entirely by humans for chimpanzees living under human care. Accordingly, these centres typically apply a slow and stepwise approach towards a life within a social group, which remains one of the most effective ways of rehabilitation [10, 49]. Little is known about how captive held chimpanzees who suffered early life adversities recover in terms of stress sensibility, social competence and how well they respond to social challenges such as changes of group composition. Therefore, we aimed at investigating how the early life experience and changes of group composition would be reflected in the social grooming networks of 18 former pet and entertainment chimpanzees living in two groups.

Our study population consisted of chimpanzees who have been confiscated from circuses and private owners and/or relocated from zoos to the primate rescue centre Fundació MONA. We focused on social grooming because it is one of the most relevant social interactions of chimpanzees [12, 50]. Grooming, beside its hygienic function [51], has important social functions such as to establish and maintain relationships, bonds and coalitions [52] as well. Additionally to promoting group cohesion and reducing tension [26, 53], grooming others also provides individual benefits as it can be traded for support in agonistic interactions [54, 55], for tolerance [56], for access to resources [57] or for food itself [58]. Furthermore several studies based on social network analysis indicate that animals with more central positions (i.e. well connected) in a network attain greater fitness/benefits than peripheral ones [59, 60]. On the contrary, in the wild a more central position might also come with the handicap of being more vulnerable to disease transmission [61, 62]. However, in a captive setting with controlled health and hygienic conditions this does not tend to be a major concern.

While high levels of self-grooming frequently have been associated with undesirable behaviours and seen as an indicator of lacking welfare [63, 64], to our knowledge high levels of social grooming have not been suggested to be a welfare issue yet. A successful integration of a new chimpanzee or the formation of a new group is, among others, often reflected in affiliative interactions with a variety of partners [65–67], rather than simply the absence of aggression [30].

Studies conducted on wild populations of chimpanzees report on grooming activities ranging from 5.7 to 11.7 percent of their daily activity budget [68–75]. However, high variations were reported for example in a study done by Wrangham [69], where grooming on average would rise up to 33 percent in artificial feeding areas.

In captivity, levels of social interaction are expected to be higher due to crowding produced by small enclosures and the absence of environmental constraints which may cause a more solitary life style in wild-living chimpanzees [67]. In captive settings with a daily changing group composition (i.e. the individuals are split into two or three parties and the composition of these parties varies every day), social grooming can rise up to 25 percent of waking hours as reported by Levé et. al. [76]. Lehmann et al. [77] reported that primates, including chimpanzees, living in the wild, tend to increase and expand their grooming activity proportionally to group size, in order to service and maintain a multitude of relationships in groups containing up to 40 individuals, but when surpassing this group size grooming activity does not increase any further. However, time spend grooming may also vary between wild-living populations due to ecological pressures such as living in harsh habitats and/or seasonality [77]. Since our study population never exceeded the number of nine individuals per group and ecological time constraints were no odds, we argue that an increased grooming activity should be seen as beneficial to the group cohesion as well as on an individual level. Thus, chimpanzees never or only rarely grooming others or directing their grooming to only one or a few group member(s) would be positioned as peripheral individuals and would thus likely have reduced benefits. Establishing and maintaining advantageous relations through grooming seems especially important when taking limitations and restrictions of a captive environment into account, where avoiding group members might be difficult at times, and tension reduction is therefore of high importance [78, 79]. Furthermore, chimpanzees in captivity tend to be less stimulated and motivated to be active compared to wild populations, due to the lack of variation, a limited enclosure size and the absence of environmental constraints [80–82]. Nevertheless, even without considering the social or hygienic functions/benefits, an increase in positive social interactions with a variety of individuals is a stimulation increment, which in captive care management tends to be a desired effect [83].

The fifth edition of the Diagnostic and Statistical Manual of Mental Disorders defines a traumatic event for children up to six years as direct or indirect exposure to the actual or threatened death, serious injury, or sexual violence or witnessing the event to a primary caregiver [84]. According to this definition, our wild-caught chimpanzees most likely experienced a traumatic life event in early infancy. Similar approaches have already been applied to studies of chimpanzees who were housed under extreme conditions such as laboratories [55, 85]. We predicted that such a traumatic experience would be reflected in the grooming networks of our wild-caught chimpanzees compared to the networks of captive born conspecifics. We are aware of the fact, that separation from the mother has detrimental effects not only on wild-caught but also on captive born infants. However, we think, that according to the descriptions found in “Visions of Caliban” [21] on how chimpanzee infants were captured from the wild, the experiences of these chimpanzees indisputably meet the criteria of a traumatic life event. Additionally, we predicted that the predominant housing condition (PHCinfant) of our study population during infancy, i.e. whether they were housed predominantly with conspecifics or without conspecifics during their first five years of life, would have an impact on their social grooming networks. We expected prolonged housing without access to conspecifics to have a negative impact on the grooming networks of the respective chimpanzees, as social grooming skills develop very slowly in infant chimpanzees [86]. Furthermore, we predicted that chimpanzees who arrived at the sanctuary at a younger age, i.e. as sub-adults, would spend more time grooming and be less choosy regarding their grooming partners compared to individuals who arrived as adults.

While early life adversities were predicted to have a long lasting effect, still detectable after being introduced and living within a social group of conspecifics for years, we assumed care management activities, i.e. alteration to the group composition, to provoke only a short-term adaptation of the individuals’ grooming interactions. To evaluate the effects of alterations of group composition on social grooming we compared the individual network measures of stable periods (i.e. periods without any changes in group composition) to those of unstable periods (i.e. periods following the introduction or separation of individuals) over a 12-year observation period. We supposed to find a difference between stable and unstable periods, as the addition of potential new allies or competitors as well as the loss of such might require a certain modification of the social strategy, though only for a short period, until already existing relationships have been reconfirmed and new ones established.

## Materials and methods

### Ethical note

This study is based purely on observational data without any invasive interventions and was conducted in accordance with all national and institutional guidelines for the care and management of primates as established by Fundació MONA, Association for the Study of Animal Behaviour/Animal Behavior Society and the Spanish Government (RD 53/2013). Any decision to alter the group composition of the chimpanzees was based on established care management protocols and at no time was influenced by research related staff members.

### Study population

The study population consisted of a total of 19 former pet and entertainment chimpanzees (seven females and twelve males in the course of the total observation time from April 2006 to July 2018) housed at the primate rescue centre Fundació MONA in Catalonia, Northern Spain. The centre is a member of the European Alliance of Rescue Centres and Sanctuaries (EARS) and it is rehabilitating chimpanzees since 2001. Behavioural observations were conducted on all of the 19 chimpanzees at MONA, but one male was never observed within a group setting of more than two individuals before being transferred to Stichting AAP, another rescue centre in Holland, and thus was excluded, resulting in a total of 18 chimpanzees included in the analysis of this study. Throughout the 12 years of observation, there were two social groups, but the number of chimpanzees housed at the centre varied due to animals passing away and new arrivals. Biographic information on the 18 individuals is shown in Table 1. Nine chimpanzees were caught from the wild, i.e. all of these chimpanzees were orphans who most probably witnessed the killing of their mother and were imported to Europe. The other nine chimpanzees were born in captivity, i.e. in an European zoo or owned by a private person. We have no information on the age at onset of maternal deprivation, neither for the wild-caught nor for the captive born individuals. We only know whether the chimpanzees were housed predominantly with or without conspecifics during their infancy, i.e. during their first five years of life. Predominantly housed with conspecifics means that the chimpanzees were housed for more than 2.5 years of their first five years of life with other chimpanzees, while predominantly housing without conspecifics means they spent more than 2.5 years without access to other chimpanzees during these first five years. Information on the exact onset, duration and sequence of the previous housing condition was not available and as such we were unable to specify this variable in more detail. As former pet and entertainment chimpanzees, all of our individuals have been socialized with humans before arriving at the rescue centre. However, we do not have detailed information on the degree or length of exposure to humans. As such, we only considered conspecifics when referring to the predominant housing condition.

**Table 1:** Characteristics and background information on the study population.

Name	ID	Sex	(Estimated) Year of Birth	Origin	Age Category at Arrival at MONA	Predominant Housing Condition During Infancy (With or Without Conspecifics)	Former Pet or Entertainment Chimpanzee*
Africa	AFR	F	2000	wild	Sub-adult	without	Pet
Bea	BEA	F	1985	wild	Adult	with	Entertainment
Bongo	BON	M	2000	captive	Sub-adult	with	Entertainment
Charly	CHA	M	1989	captive	Sub-adult	with	Entertainment
Cheeta	CHE	F	1990	wild	Adult	without	Entertainment
Coco	COC	F	1994	wild	Adult	without	Pet/ Entertainment
Juanito	JUA	M	2003	captive	Sub-adult	with	Pet/ Entertainment
Marco	MAR	M	1984	captive	Adult	with	Entertainment
Nico	NIC	M	2001	captive	Sub-adult	without	Pet/ Entertainment
Pancho^1^	PAN	M	1990	captive	Sub-adult	with	Entertainment
Romie^2^	ROM	F	1979	wild	Adult	with	Entertainment
Sara^3^	SAR	F	1998	captive	Sub-adult	without	Pet/ Entertainment
Tico	TIC	M	1985	wild	Adult	without	Entertainment
Tom	TOM	M	1985	wild	Adult	with	Entertainment
Toni	TON	M	1983	wild	Adult	with	Entertainment
Toto^4^	TOT	M	1956	wild	Adult	with	Entertainment
Victor	VIC	M	1982	captive	Adult	without	Pet/ Entertainment
Waty	WAT	F	1996	captive	Sub-adult	with	Entertainment

Abbreviations: F = female, M = male.

^1^died in 2007,

^2^died in 2011,

^3^died in 2012,

^4^died in 2013.

*Chimpanzees often were used for several purposes before arriving at the rescue centre. The term "Entertainment" refers to any type of commercial use such as tourist attraction, street performing, media performing, circus performing and instances of zoo housing.

Wild-caught chimpanzees typically arrived as adults (except for one adolescent individual) at Fundació MONA (mean age: 23.6 ± 10.4 years), whereas the captive born subjects were mostly sub-adults (except for two adults) upon arrival (mean age: 9.2 ± 7.5 years). Considering that our wild-caught chimpanzees have on average spent more time in the pet and entertainment business than captive born ones, we included the age at arrival at the sanctuary as another variable describing the chimpanzees past. Thus, we assigned all chimpanzees to the respective age category, i.e. adult or sub-adult, at their time of arrival at Fundació MONA.

The chimpanzees were living in one of the two different social groups (Mutamba group, Bilinga group). Over the 12-year observation period, several alterations to the group composition occurred in order to integrate new chimpanzees, transfer animals between groups for welfare reasons or due to the natural death of individuals. The alteration of a group composition is defined as the change of the minority of the individuals of a group by either adding or removing one or several individuals at a time. Within the 12 years of data collection, the group sizes could vary from a minimum of four to a maximum of nine individuals, but was most of the time between five to eight individuals per group (Table 2).

**Table 2:** Chronology of the different observation time periods for the two social groups, Mutamba (M1-M6) and Bilinga (B1-B12), included in this study.

Observation time period	Observation phase	Group size	Individuals	Group composition	Explanatory note
M1	2006–2007	4	Mar, Cha, Ton, Pan	stable	
M2	2008	4	Mar, Cha, Ton, Bon	unstable	Bon just joined the group
M3	2008–2011	4	Mar, Cha, Ton, Bon	stable	
M4	2012–2017	5	Mar, Cha, Ton, Bon, Jua	stable	
M5	2017	7	Mar, Cha, Ton, Bon, Jua, Afr, Wat	unstable	Afr and Wat just joined the group
M6	2018	7	Mar, Cha, Ton, Bon, Jua, Afr, Wat	stable	
B1	2006–2007	7	Tot, Rom, Wat, Bon, Sar, Jua, Nic	stable	
B2	2010	9	Tot, Rom, Wat, Sar, Jua, Nic, Tic, Vic, Afr	unstable	Integration of Afr
B3	2010	9	Tot, Rom, Wat, Sar, Jua, Nic, Tic, Vic, Afr	unstable	Afr just joined the group
B4	2011	8	Tot, Wat, Sar, Jua, Nic, Tic, Vic, Afr	stable	
B5	2011	7	Tot, Wat, Sar, Nic, Tic, Vic, Afr	unstable	Jua just left the group
B6	2012	6	Tot, Wat, Nic, Tic, Vic, Afr	unstable	Integration of Tom
B7	2012	6	Tot, Wat, Nic, Tic, Vic, Afr	unstable	Integration of Tom, Coc, Bea
B8	2013	5	Wat, Nic, Tic, Vic, Afr	unstable	Right after passing of Tot
B9	2013–2015	8	Wat, Nic, Tic, Vic, Afr, Tom, Coc, Bea	unstable	Integration of Tom, Coc, Bea
B10	2017	9	Wat, Nic, Tic, Vic, Afr, Tom, Coc, Bea, Che	unstable	During fusion of Wat, Nic, Tic, Vic, Afr and Tom, Coc, Bea, Che
B11	2017	7	Nic, Tic, Vic, Tom, Coc, Bea, Che	unstable	After separation of Wat, Afr
B12	2018	7	Nic, Tic, Vic, Tom, Coc, Bea, Che	stable	

Reasons for the gaps in between the observation phases are explained in the Data sampling section

Observations were conducted only while the chimpanzees had access to a naturalistic outdoor enclosure (5 640 m^2^), equipped with a multitude of climbing structures, which give them the opportunity to exploit natural and artificial resources. The enclosure is divided into two separate areas to accommodate both groups: the first of 3 220 m^2^ and the second of 2 420 m^2^, with a total perimeter of 191 m. A steel mesh and an electrified fence surround the enclosure. For more detailed information on the outdoor enclosure see [87, 88].

The chimpanzees are fed four times per day with a balanced diet based on fruits, seeds and vegetables. They have limited quantities of other protein-rich foods (constant since 2001) and have access to water *ad libitum*. A big portion of their daily diet is scattered and hidden in the outdoor enclosures to stimulate natural foraging behaviour and locomotion as part of their daily enrichment program.

### Data sampling

Data on the chimpanzees’ behaviour was recorded over 146 months from April 2006 to July 2018 by using instantaneous scan sampling [89] every two minutes for all individuals of one group in view. Data was recorded between approximately 1030 hours and 1830 hours while the chimpanzees had access to the outdoor enclosure. Observation sessions of 20 minutes were evenly distributed between mornings and afternoons on randomised days (Monday to Sunday). Observers were only permitted to collect data after the completion of an observation training period and successfully passing the inter observer reliability test (agreement ≥85%) with the head of research at the centre (M. Llorente).

Although a complete set of behaviours was recorded, for this study we only considered "social grooming given" to group members. For this purpose, we created a directed grooming matrix for each group composition within each observation time period (see Table 2). More precisely, we calculated the percent of scans where individual A groomed individual B within a certain group composition and observation time period. To this end, we divided the number of scans where individual A was grooming B by the number of scans both individuals spent together in the outdoor enclosure and had access to each other, and multiplied the quotient by 100. These calculations were done for every individual of the two social groups for every group composition and observation time period. A total of 303 123 scans have been filtered for "grooming given" and used for this study (Bilinga group 197 053; Mutamba group 106 070). Slight modifications to the behavioural catalogue have occurred between 2006 and 2018, however, none of them affecting the validity of the grooming records. Observers recorded social grooming including the sender and receiver of each grooming interaction, following previous research carried out at the sanctuary [10].

Over the 12-year observation period we identified a total of 42 time periods where it was possible to define a clear ‘stable’ or ‘unstable’ condition produced by alteration of the group composition. A time period was labelled as ‘stable’ when group composition did not change for at least four months beforehand and did not experience any short- or long-term changes to its composition. A time period was labelled as ‘unstable’ when a permanent composition alteration (removal or addition of an individual) or frequent short-term changes of group composition due to active integration activities (process of adding a new individual) have occurred, for a minimum duration of four months. However, not all the data available could be included due to a lack of records or unevenly distributed observation sessions during certain time periods. Therefore, we selected 18 observation time periods (seven stable and 11 unstable) for this study. We excluded the rest due to an insufficient amount of observations (not reaching a minimum of 480 scans per individual and time period, which corresponds to 16 hours or two full days of observation) or an uneven distribution of the observation sessions within a time period.

For all of the 18 chimpanzees the percent of scans every single individual is grooming each of its group members in any of these 18 time periods are used for further analysis. The number of different time periods per chimpanzee varied from one to 12, varying among others due to the difference in the year of arrival.

The percent of scans an individual spent on grooming a group member per time period and group composition was used to calculate two individual social network measures to evaluate the standardized grooming activity of every chimpanzee and his choosiness in the distribution of grooming among group mates. This added up to 119 data points for the standardized grooming activity and 104 data points for the distribution of grooming as distribution of grooming could only be calculated for individuals who were grooming at least one group member in the respective observation time period.

### Social network analysis (SNA)

Most studies on chimpanzees define the social networks by scoring dyadic grooming interactions [44, 76, 90], or by recording information of dyadic spatial association [43]. Due to the database available for this study and based on the fact that grooming is an important social behaviour in chimpanzees [52] we created matrices of directed dyadic grooming interactions obtained for each time period and group composition.

We created our networks in R environment [91] using Igraph 0.5.5–3 [92] for visual representation of the graphs. R script was adapted according to McFarland et al. [93]. The weighted network graphs consist of nodes representing the individuals and directed edges representing the percent of scans an individual spent grooming its group members (S1 Fig).

The grooming matrices were used to calculate the following two network measures which have been previously described by Kasper and Voelkl [94] and used by Kalcher-Sommersguter et al. [27].

#### VSC: The vertex strength centrality

The vertex strength centrality $C_{s}(v_{i})=\frac{s_{i}}{N-1}$ is a measure to describe the standardized strength of an individual’s grooming activity. More precisely, it reflects the mean percent of scans an individual spent grooming another individual of his group, while taking the group size into account. It is being calculated by dividing the vertex strength *s*_*i*_ by the number of group members *-1 (N-1)*. The vertex strength *s* of vertex *i* is given by $s_{i}=\sum_{j=1}^{N}w_{ij}$, *w* being the corresponding weight of the edges connected to a vertex.

#### DEWD: Deviation from edge weight disparity

The edge weight disparity $Y_{2}(v_{i})=\sum_{j=1}^{N}(\frac{w_{ij}}{s_{i}}{)}^{2}$ is a measure reflecting how evenly an individual is distributing his grooming among all group members. This value ranges from 1/(N-1) to 1, with 1/(N-1) representing a perfectly even distribution of grooming among all possible group members, higher values representing a more restricted distribution of grooming among group members and 1 means that all grooming is given towards one single group member. By calculating the deviation from this edge weight disparity *(Y*_*2*_*(v*_*i*_*))* we are able to compare the distribution of grooming between groups of different group size. This is being obtained by calculating the equal disparity *Y*_2_ per group which is 1/(N-1) and computing the deviation from *Y*_*2*_ for each individual by subtracting the group specific *Y*_*2*_ from the individual *Y*_*2*_*(v*_*i*_*)*. Thus, the deviation from the edge weight disparity ranges from 0 to (N-2)/(N-1), where 0 represents a perfectly even distribution of grooming among all possible group members and (N-2)/(N-1) represents the total concentration of all grooming given towards one single group member. Note that the deviation from edge weight disparity could only be calculated for individuals who were grooming at all.

The summations in the formulas of vertex strength and edge weight disparity include the edges extending from vertex *i* to all vertexes other than *i* (i.e. with *j* ≠ *i*).

### Statistical analysis

The effects of various factors on the two individual weighted network measures, vertex strength centrality (VSC) and deviation from edge weight disparity (DEWD) were assessed using linear mixed models (LMMs). Time-Period-Stability (i.e. stability of group composition: stable vs. unstable), Arrival-Age-Category (i.e. the age category at arrival at the sanctuary: sub-adult vs. adult, according to Goodall [12]), Sex (female vs. male), Origin (wild-caught vs. captive born) and PHC-infant (i.e. predominant housing condition during infancy: predominantly housed with conspecifics vs. predominantly housed without conspecifics) were fixed factors in our full models. We examined the multicollinearity between fixed factors by calculating the variance inflation factor (VIF). Individual IDs and Observation-Time-Period (i.e. a code for the respective group composition in a certain time period; Table 2) were random factors. We included the Observation-Time-Period as a random factor as observations extended over a 12-year time period and could have been influenced by other unknown factors not recorded in our data.

We used multi-model interferences to compare and rank all 33 possible candidate models (including the Null-model) according to their respective *AICc* (Akaike Information Criterion after correction for small sample sizes) for DEWD and VSC models, respectively. Thus, all fixed factor combinations were taken into account and models were ranked according to the lowest/best *AICc*. We further calculated the *ΔAICc* and the *AICw* (normalized Akaike weights) for all candidate models.

Model interference and selection was performed using model averaging [95]. We based the selection of the subsets of best models on the *ΔAICc* and considered all models with a *ΔAICc* lower than 10 compared to the best model as equally possible candidates. We further indicate the *RVI* (relative variable importance) of all fixed factors of the averaged model, which is calculated as a sum of all *AICw* over all subset models that include the respective fixed factor. Model fit was assessed via graphical evaluation of the residuals (S2–S5 Figs).

In a next step, we created models based on the full models with now additionally including interactions of previously found significant fixed factors with high RVI scores. We analyzed the significance of said interactions via Type III analysis of variance (ANOVA) using the Satterthwaite approximation for degrees of freedom.

Post hoc Type III analysis of variance (ANOVA) was performed using the Satterthwaite approximation for degrees of freedom. We ran Linear Mixed Models (LMMs) using the "lme4" package [96] and all related analysis, such as the VIF calculations using the "car" package [97] and Model Averaging using the "MuMln" Package [98] in the R environment [91].

## Results

We calculated two different social network measures (i.e. the vertex strength centrality and the deviation from edge weight disparity) for each individual per time period and group composition, and ran LMMs to investigate the effects of an (un-)stable group composition, arrival age, sex, origin and predominant housing condition during infancy on social grooming networks. The vertex strength centrality (VSC) represents the standardized strength of an individual’s grooming activity. The deviation from edge weight disparity (DEWD) reflects an individual’s choosiness/restriction in his distribution of grooming among its group members. As the deviation from edge weight disparity is a value limited between 0 and (N-2)/(N-1) we applied a logit transformation. However, the transformation did not lead to any changes regarding the model selection and outcomes. Therefore, we decided to discuss the results of the original DEWD models without logit transformation.

For model selection, we chose the model averaging approach, which means that not only the best model (with the lowest *AICc*) is considered, but also models with a *ΔAICc* lower than 10 compared to the best model. These subsets including all model candidates with a *ΔAICc* lower than 10 are listed in the supplementary material (S1 and S2 Tables).

The variance inflation factor (VIFs) for the five fixed factors of our final full models ranged between 1.02–1.77 indicating that our fixed factors were not correlated.

All post hoc tests were conducted on the full models as all five fixed effects were retained within the averaged subsets.

### Effects on chimpanzees´ grooming activity

**Fig 1:**
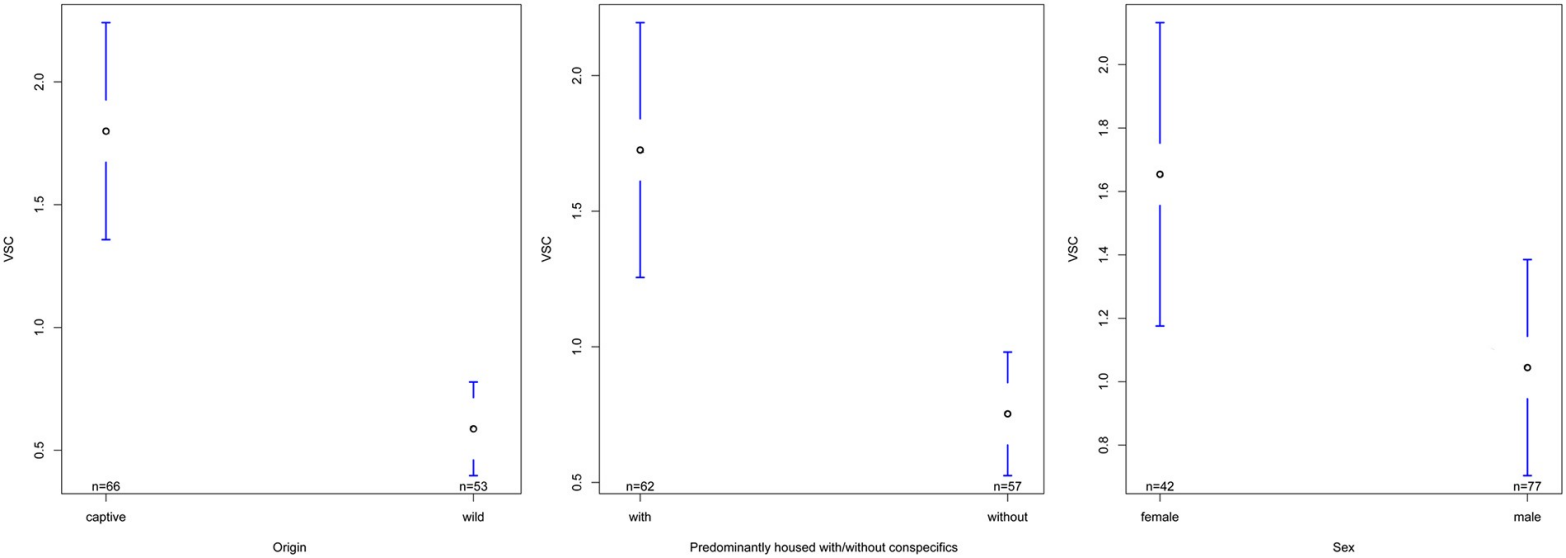
Confidence Interval plots of VSC and all significant fixed factors (Origin, PHCinfant, Sex). Mean vertex strength centrality (±95% CI).

The standardized grooming activity of our chimpanzees, i.e. the VSC, was significantly influenced by Origin, Predominant housing condition during infancy (PHCinfant), and Sex (Table 3). With respect to Origin we found captive born chimpanzees (*N* = 66 data points) to have a significantly higher grooming activity than wild-caught chimpanzees (*N* = 53 data points; Fig 1). The factor Origin had a strong influence on the grooming activity indicated by a high relevant variable importance of *RVI*_*VSC*_ = 0.99 (Table 3) and ANOVA *post hoc* testing showed Origin to be a significant predictor influencing vertex strength centrality (*F*_1,11_ = 15.52, *P*<0.01, S3 Table). The factor Predominant housing condition during infancy had a relatively high relevant variable importance of *RVI*_*VSC*_ = 0.86 (Table 3). We found that chimpanzees who were predominantly housed with conspecifics during infancy (N = 62 data points) groomed their group mates significantly more than chimpanzees who were predominantly housed without conspecifics during their first five years of life (N = 57 data points; Fig 1; ANOVA *post hoc* test: *F*_1,15_ = 8.03, *P*<0.05; S3 Table). With respect to Sex we found females (*N* = 42 data points) to have a significantly higher grooming activity than males (*N* = 77 data points; Fig 1). The factor Sex had a high variable importance of *RVI*_*VSC*_ = 0.92 (Table 3). An ANOVA *post hoc* test (*F*_1,15_ = 11.98, *P*<0.01) showed that Sex was a significant predictor influencing the VSC (S3 Table).

**Table 3:** Averaged best vertex strength centrality (VSC) model and relative importance of the fixed effects.

**VSC Model: conditional average (ΔAIC<10)**					
	Estimate	Std. Error	Adjusted SE	z value	Pr(>|z|)
(Intercept)	2.198	0.514	0.518	4.243	<0.001 ***
Sex	-1.045	0.317	0.321	3.257	0.001 **
PHCinfant	0.840	0.301	0.304	2.762	0.006 **
Origin	-1.362	0.337	0.340	4.002	<0.001 ***
TPstability	-0.226	0.435	0.440	0.513	0.608
ArrivalAgeCat	-0.093	0.420	0.424	0.220	0.826
**Relative variable importance**:					
	Origin	Sex	PHCinfant	TPstability	ArrivalAgeCat
Importance:	0.99	0.92	0.86	0.26	0.26
N containing models:	14	8	8	6	8

Output of the averaged best vertex strength centrality (VSC) model and relative importance of the fixed effects sex, predominant housing condition during infancy (PHCinfant), origin, arrival age category (ArrivalAgeCat) and stability of group composition (TPstability). All models included in this averaged results are presented in the supporting material (S1 Table). Signif. codes:

‘***’ ≤0.001

‘**’ ≤0.01

‘*’ ≤0.05

‘.’ ≤0.1

‘ ’ ≤1

The factors Arrival age category (ArrivalAgeCat) and Stability of group composition (TPstability) were all retained variables in the best model selection but had a very low relative variable importance (*RVI*_*VSC*_ = 0.26) and failed to show any significant effect on the grooming activity (Table 3).

**Fig 2:**
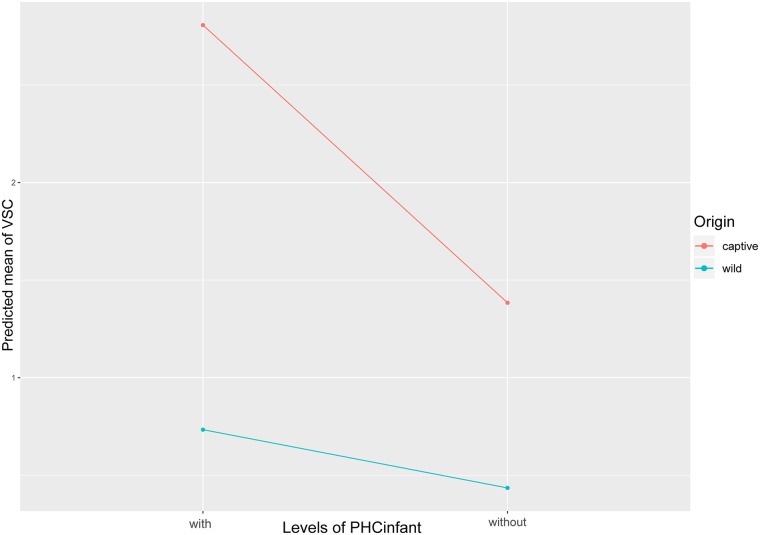
Plot representing the effect of the interaction between Origin and PHCinfant on the grooming strength (VSC). Each point on the plot is a predicted mean VSC value and each connection of two points describes the effect, based on the data of the VSC LMM model with TPstability, Origin, Sex, Arrival Age Category, Predominant Housing Condition during Infancy and the interaction of Origin and PHCinfant as fixed factors.

Considering that both, Origin and the Predominant housing condition during infancy, significantly affected the grooming strength (VSC) in our VSC model, both with high RVI scores, we ran a separate VSC model based on the full model but added the interaction of these two fixed factors. Due to slightly elevated VIFs (2.1–3.1) detected between the interaction and the separate components (Origin and PHCinfant) we ran this LMM apart from the averaged LMM. We found the interaction to have a significant impact on the grooming strength (*F*_1,13_ = 4.90, *P*<0.05; S4 Table). The plot (Fig 2) shows, as already indicated in our averaged VSC model, that (a) captive born chimpanzees had a higher grooming activity than wild-caught chimpanzees, and (b) chimpanzees that were predominantly housed with conspecifics had a higher grooming activity than those predominantly housed without conspecifics during infancy. The interaction plot, however, also shows, that both, captive born chimpanzees predominantly housed with conspecifics and those predominantly housed without conspecifics during infancy, spent significantly more time on grooming given compared to wild-caught chimpanzees irrespective of their predominant housing condition during infancy.

### Effects on chimpanzees´ grooming distribution

**Fig 3:**
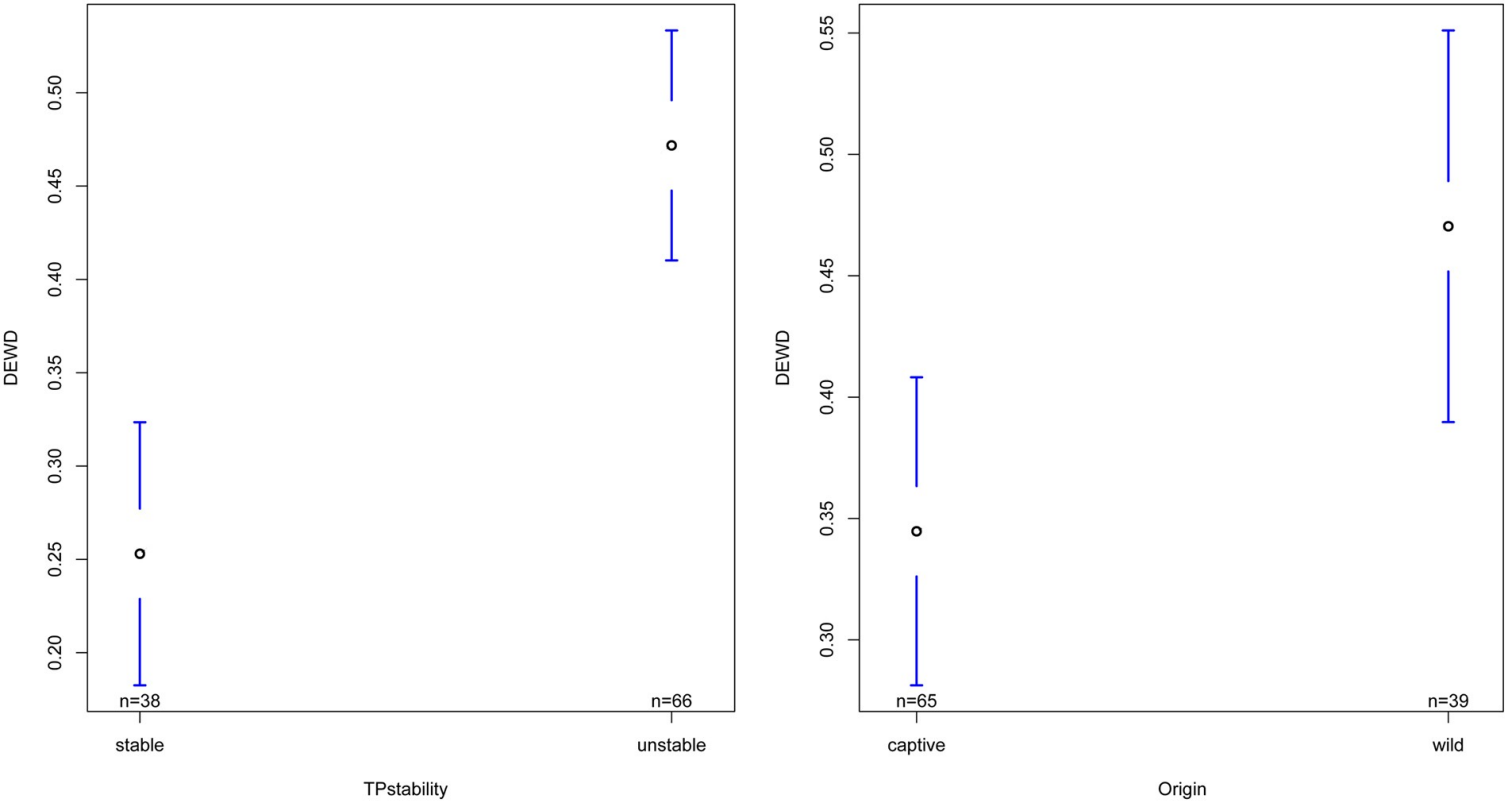
Confidence interval plots of DEWD and both significant fixed factors, Origin and TPstability. Mean deviation from edge weight disparity (±95% CI).

The individuals’ choosiness/restriction in their distribution of grooming, i.e. the DEWD, was significantly influenced by Origin and Stability of group composition (TPstability) (Table 4). With respect to Origin we found captive born chimpanzees (*N* = 65 data points) to have a significantly lower deviation from edge weight disparity than wild-caught chimpanzees (*N* = 39 data points; Fig 3). That means that captive born chimpanzees distributed their grooming much more evenly among group mates than did wild-caught chimpanzees who were more restricted in whom they were grooming. Origin had a strong and significant influence with a relatively high relevant variable importance of *RVI*_*DEWD*_ = 0.83 (Table 4). A ANOVA *post hoc* testing showed Origin to be a significant predictor influencing deviation from edge weight disparity (*F*_1,7_ = 9.60, *P*<0.05; S5 Table). Stability of the group composition (TPstability) proved to be a very important factor with a *RVI*_*DEWD*_ = 0.97 (Table 4). The chimpanzees distributed grooming significantly more evenly among their group mates during stable periods, i.e. periods without any alteration to the composition of the group (*N* = 38 data points), compared to unstable periods (*N* = 66 data points; Fig 3). A ANOVA *post hoc* test (*F*_1,15_ = 10.15, *P*<0.01; S5 Table) supported this finding.

**Table 4:** Averaged best deviation from edge weight disparity (DEWD) model and relative importance of the fixed effects.

**DEWD Model: conditional average (ΔAIC<10)**					
	Estimate	Std. Error	Adjusted SE	z value	Pr(>|z|)
(Intercept)	0.165	0.103	0.104	1.586	0.112
TPstability	0.214	0.066	0.067	3.199	0.001 **
Sex	0.113	0.061	0.061	1.831	0.067 .
Origin	0.153	0.062	0.063	2.433	0.015 *
PHCinfant	0.056	0.057	0.058	0.973	0.331
ArrivalAgeCat	0.022	0.077	0.077	0.287	0.774
**Relative variable importance**:					
	TPstability	Origin	Sex	PHCinfant	ArrivalAgeCat
Importance:	0.97	0.83	0.55	0.34	0.30
N containing models:	16	15	12	11	11

Averaged best deviation from edge weight disparity (DEWD) model and relative importance of the fixed effects sex, predominant housing condition during infancy (PHCinfant), origin, arrival age category (ArrivalAgeCat) and stability of group composition (TPstability). All models included in this averaged results are presented in the supporting material (S2 Table). Signif. codes:

‘***’ ≤0.001

‘**’ ≤0.01

‘*’ ≤0.05

‘.’ ≤0.1

‘ ’ ≤1

The factors Predominant housing condition during infancy (PHCinfant), Arrival age category (ArrivalAgeCat) and Sex were retained variables in the best model selection, but scored low to medium in their relevant variable importance with *RVI*_*DEWD*_ = 0.30–0.55 and did not demonstrate any significant influence on the grooming distribution among group mates (Table 4).

**Fig 4:**
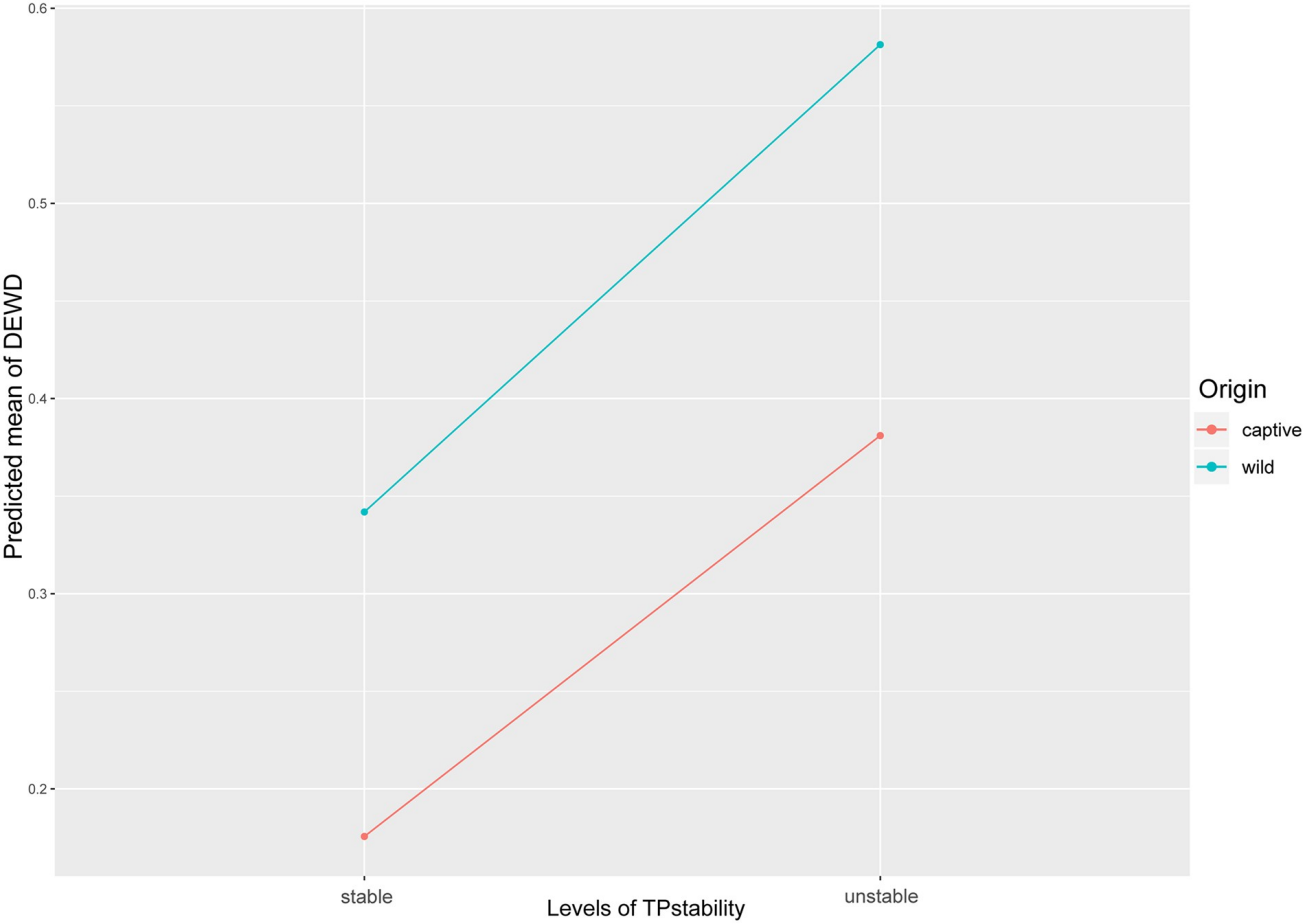
Plot representing the effect of the interaction between Origin and TPstability on the grooming distribution. Each point on the plot is a predicted mean DEWD value and each connection of two points describes the effect, based on the data of the DEWD LMM model with TPstability, Origin, Sex, Arrival Age Category, Predominant Housing Condition during Infancy and the interaction of TPstability and Origin as fixed factors.

Considering that both, Origin and Stability of the group composition, significantly affected the distribution of grooming in our DEWD model, both with high RVI scores, we decided to run an additional DEWD model, based on the full model containing all five previously described fixed factors and added the interaction between TPstability and Origin. Due to slightly elevated VIFs (1.2–3.4) detected between the interaction and the separate components (TPstability and Origin) we ran this LMM apart from the averaged LMM. We found no significant result for the interaction (S6 Table). Nevertheless, we show a figure, to be able to visualize and discuss the tendencies of the interaction between Origin and TPstability (Fig 4). Here we can see, that (a) captive born chimpanzees distribute their grooming more evenly among their group mates than wild-caught chimpanzees, (b) unstable time periods are characterized by a more restricted grooming distribution compared to stable periods, and (c) both, captive born and wild-caught chimpanzees, distribute their grooming more evenly among group members during stable periods compared to unstable periods. Hence, captive born and wild-caught chimpanzees seem to react in a very similar way to alterations of the group composition, i.e. restricting the grooming distribution during unstable periods and grooming more evenly distributed during stable periods.

Graphical representations of the weighted social grooming networks of all 18 Observation Time Periods/Group compositions for both chimpanzee groups are presented in chronological order in the supporting information (S1 Fig). These networks also show that there is one male, Tico (TIC), who was observed grooming only in one out of a total of eleven time periods. Tico is a wild-caught male who was predominantly housed without conspecifics during his infancy.

## Discussion

For this study, we chose two individual social network measures in order to describe the social grooming networks and the individuals’ social capacities: the standardized strength of grooming given and the distribution of grooming given among group mates. The findings of our study show that early life adversity is reflected in the social grooming networks of former pet and entertainment chimpanzees. Two out of the three factors referring to past experiences/conditions, i.e. Origin and Predominant housing conditions during infancy, significantly affected one or both social network measures. With respect to Origin, we found that the wild-caught chimpanzees of our study groups spent less time on "grooming given" and were much more restricted in the selection of grooming partners compared to captive born ones. Regarding Predominant housing conditions during infancy, we could show that chimpanzees who were housed predominantly with conspecifics spent more time grooming their group members compared to individuals that were housed predominantly without conspecifics during infancy. Relating to the third factor, Age at arrival at the rescue centre, we did not find any differences between chimpanzees who arrived as sub-adults and those arriving as adults.

Beyond that, alterations to the composition of the group had an impact on social grooming, as the distribution of grooming among group mates was significantly more restricted during the unstable periods where alterations occurred, compared to stable periods without any alterations to the group composition.

### Long lasting influence of early life adversities on social capacities

It is known that chimpanzees are highly social animals who create and live in complex social networks [99]. In order to develop normally in these complex social systems, chimpanzees learn from their mother and group mates from an early age [12]. An early environment satisfying the needs for security and exploration as provided by the mother and access to social partners are essential to develop the necessary social skills and become socially competent. This environment is especially important during the first two to three years of life, when infant chimpanzees tend to be inseparable from their mother [11].

By comparison, none of the chimpanzees of our study population did grow up under species-appropriate conditions. While most of the former pet chimpanzees grew up with humans only, the majority of the former entertainment chimpanzees were at least partially socially reared but were dressed and trained to perform for instance in a circus. Bloomsmith et al. [100] showed that social isolation and sensory deprivation during the first years of an infant’s life has a negative impact on the ability to live in social groups. Some studies even suggest that traumatic early life events could lead to mood and anxiety disorders in chimpanzees which, among others, become obvious in no or little interest in social interactions with conspecifics [101].

In order to gain a central position within a social group it is necessary to invest energy and time in social bonding activities, in chimpanzees this is often achieved through grooming interactions. Thus, lacking social experience and skills might reduce the capacity and/or the desire to engage in social grooming interactions and result in the inability to manage multiple relations at any one time. As such, this reduced social activity was found to have a negative impact on the individual’s social position and possibly welfare in early maternally and socially deprived former laboratory chimpanzees who were all caught from the wild [102].

While we know that all of the chimpanzees of our study sample had harmful experiences in the past and most were separated from their mothers at an early age, we nevertheless believe that the chimpanzees who were caught in the wild had an additional traumatic experience. This traumatic experience consisted of witnessing the killing of their mother and other group members, capture and transportation under extreme and often harmful conditions, as well as a dramatic change in their living conditions from wild to captivity. Although all this occurred in a relatively short amount of time, the extreme conditions, potential injuries and stress experienced by these infants during the most vulnerable period of early infancy often led to death during capture and transportation [103, 104]. As for those who survived, we expected said traumatic experiences to be reflected in their social grooming activity, even after living in a social group for years. Indeed, we found our wild-caught chimpanzees to engage less in social grooming and to be more selective in whom they groomed compared to our captive born chimpanzees. Furthermore, individuals who were completely isolated from the grooming network in some of the observation periods, i.e. individuals who did not groom other chimpanzees at all, were mainly wild-caught ones. Interestingly, a recent study [105] did not find an effect of origin on social grooming. This might be explained by the fact that only frequency, but not duration of grooming was taken into account and no differentiation was made between grooming given and received.

The majority of our wild-caught chimpanzees arrived as adults (mean age: 23.6 ± 10.4 years) and most of the captive born individuals were sub-adults (mean age: 9.2 ± 7.5 years) upon arrival at the sanctuary. We were aware of the fact that this difference in age might be a confounding variable affecting origin. Therefore, we also tested for a possible effect of age at arrival at the rehabilitation centre. In view of this, it was surprising, that we did not find any effect of age at arrival at the rehabilitation centre, neither in the chimpanzees’ grooming activity nor in their distribution of grooming among group members. This finding suggests that the significant difference in grooming between wild-caught chimpanzees, who by majority arrived as adults, and captive born chimpanzees, who by majority arrived as sub-adults, seems not to be due to the age at arrival at the rescue centre but due to the origin of the chimpanzees. However, we do believe that an older age at arrival at the rescue centre might result in more difficulties during the initial integration process compared to a younger one. Furthermore, the arrival age category, as the name indicates, only refers to the time the chimpanzees arrived at the sanctuary and started their rehabilitation and social integration into one of the two existing groups. However, several of the individuals were not transferred directly to the sanctuary after their time as pets or entertainment animals, but instead spent some time (in some cases years) without being forced to train or perform or were temporarily relocated to zoological gardens, before arriving at the sanctuary. These in between housing situations were at times marked by improved living conditions, as in some cases the chimpanzees were housed with conspecifics and/or received species-appropriate care.

Since Kalcher-Sommersguter et al. [102] could show that early socially deprived wild-caught former laboratory chimpanzees spent significantly less time on "grooming given" compared to later deprived ones, we tested for predominant housing conditions during infancy and expected chimpanzees predominantly housed without conspecifics during infancy to be more restricted and less active groomers compared to those housed predominantly with conspecifics. We did not find any significant differences regarding the distribution of grooming, but our results indicate that chimpanzees predominantly housed with conspecifics during infancy spent more time grooming others than those predominantly housed without conspecifics.

One possible explanation for this finding might be related to the amount of human exposure. Although we do not have detailed information on our study subjects with respect to the amount of human exposure, it seems very likely that chimpanzees predominantly housed without conspecifics during infancy had more interactions with humans compared to those predominantly housed with conspecifics. The social interaction with one or several humans might have helped to develop certain social skills. However, as humans usually do not use grooming to interact with chimpanzees, the slowly developing grooming behaviour [86] might not have received a sufficient amount of practice opportunities and reinforcement during infancy in the chimpanzees living without conspecifics. Freeman & Ross [4] reported that chimpanzees who experienced more exposure to conspecifics and less exposure to humans during their first four years of life showed the most grooming. Furthermore, Jacobson et al. [106] found that chimpanzees with more exposure to humans during their early life had higher levels of cortisol, indicating elevated stress levels. In this line, a study looking into the relationship between social behaviours and hair cortisol concentrations, reported that rhesus macaques who spent more time socially active with conspecifics had significantly lower levels of cortisol [107]. Regarding our results and these findings, we argue that the lower grooming activity of our wild-caught chimpanzees and those predominantly housed without conspecifics during infancy has to be seen as a social limitation, which is potentially resulting in higher levels of stress and a reduced wellbeing.

The interaction between Origin and the Predominant housing condition during infancy on the grooming strength revealed that while captive born chimpanzees generally exhibited a far higher grooming activity than wild-caught chimpanzees the former also seemed to be affected more strongly by the Predominant housing condition. This became visible through a steeper decline in the grooming activity between captive born chimpanzees predominantly housed with conspecifics and those housed predominantly without conspecifics compared to the much smaller decline between wild-caught chimpanzees housed predominantly with conspecifics and those housed predominantly without conspecifics.

In a study conducted by Kalcher-Sommersguter et al. [27], the same social network measures were applied to compare the social grooming networks of ex-laboratory and zoo chimpanzees. Mother-reared zoo chimpanzees were found to distribute their grooming evenly among group mates, whereas wild-caught zoo chimpanzees who were maternally deprived but socially reared in their first two years of life were restricted in their distribution of grooming among group members similar to what we found in our wild-caught former pet and entertainment chimpanzees.

According to our findings, we suggest considering information on the animals´ life history, such as the origin and the predominant housing condition during infancy, when introducing new individuals into already existing groups and during the formation of new groups.

### The influence of sex on individual social network measures

Even though the focus of our study was on social conditions, we found sex to have an impact on the social grooming networks as well. Lehmann and Boesch [108] suggest that even though previous studies on wild chimpanzees indicated males to be more socially active than females, this might depend greatly on the dispersal pattern and habitat quality. Moreover, it has been shown that the social potential of females becomes apparent in captive settings where competition for resources is less of an issue [109]. This is in line with our findings as the grooming activity was even higher in females compared to males and females did not differ from males regarding their distribution of grooming among group mates.

### Short-term reactions to alterations of group composition

Living in a social group cannot be compared directly between wild ranging populations and groups living in captivity [54] due to significant differences in living conditions such as food availability, medical care, etc. However, it has to be expected that the significance of being able to establish and maintain social relationships with other group members and to perform complex social interactions holds not only for individuals living in the wild [110, 111] but also for those in captivity. Nevertheless, social activity might also cause a certain amount of stress and becomes even more demanding when changes to the group composition occur or new unfamiliar individuals join a group [48]. We were able to study the adaptation of individuals to group alterations, i.e. during social challenging time periods, by comparing observation periods with alterations in group composition (unstable periods) to periods without alterations (stable periods). As such, we were able to demonstrate, that chimpanzees changed their social strategy during unstable periods compared to stable periods, by modifying their distribution of grooming among group members, i.e. by abandoning or weakening certain bonds while strengthening others or forming new ones without changing the amount of "grooming given".

Our findings show that unstable periods were characterized by a more selective choice of grooming partners. It is important to note, that we refrain from labelling this mentioned adaption, i.e. the change in the distribution pattern, as either positive or negative, but argue that this indicates a certain capacity to react to a social alteration. More importantly, this adaptation seems to be shown by all of our chimpanzees, regardless of their level of choosiness/restriction. Interestingly, the individuals did not differ in the time they spent on "grooming given" between stable and unstable periods, but seemed to be choosier during unstable periods compared to stable periods.

One possible explanation for this might be that the amount of grooming needed to maintain a position during stable periods is similar to the amount of the, then more unevenly distributed, grooming needed to form new relationships beside the maintenance of already formed bonds during unstable periods. Another possible explanation might be that the individual’s grooming activity in stable periods already represents the maximum amount an individual is willing to engage. We can exclude the possibility that adding new individuals produced the more uneven distribution of grooming during unstable periods, as unstable time periods include not only the addition of group members, but also reductions in group size and changes to the routine (temporarily splitting and shuffling of a group).

One important finding of our study is that the changes in the distribution of grooming among group mates during unstable periods did not last permanently. This became apparent by the fact that unstable periods alternated with stable periods during the 12-years of observations (see Table 2) and the distribution pattern differed clearly between stable and unstable periods.

Due to the interaction plot between Origin and Time period stability we could further see, that regardless of the differences in origin, the chimpanzees modified their grooming distribution similarly. This means that, at least under attentive caring conditions, provided for example by a sanctuary such as Fundació MONA, even individuals with adverse early life experiences detect and react to socially challenging situations such as group alterations, by adjusting and after a few month slowly readjusting their grooming distribution pattern.

We do know that the socio-emotional development is a complex process and retrospective studies focusing on early life history include several risks, which can easily lead to misinterpretations. The relatively small sample size of 18 individuals and the vague information on the early life history and pre-sanctuary experience might have caused that we overlooked other potentially important factors. However, the observation period of 12 years and detailed information on the group management allowed us to analyse the effects of alterations to the group composition in detail. By no means do we suggest that the factors, considered here, are the only factors influencing the social grooming of these study population, but rather wish to emphasise that diverse factors and possibly their combinations could have a potentiating or moderating effect.

The wild-caught chimpanzees of our study population are on average older than the captive born chimpanzees and as such, most of the wild-caught individuals arrived at an older age at the sanctuary compared to the captive born ones. We considered this in our analysis and found origin but not age at arrival to influence the chimpanzees’ grooming activity. A study conducted on former laboratory chimpanzees has shown that the age at onset of deprivation but not the age at observation time and the years spent in deprivation accounted for differences found in social behaviour [9], supporting our finding. However, to be able to clearly disentangle the effects of origin and age at arrival at the sanctuary would require a study population consisting of wild-caught and captive born individuals in the same age ranges and with detailed information on every individual’s life history.

In conclusion, we could show that early traumatic life events and adverse living conditions during infancy, affect the social grooming of former pet and entertainment chimpanzees in the long term. Wild-caught individuals spent less time on grooming given and were more restricted in whom they groomed compared to captive born ones, and chimpanzees who have been predominantly housed without conspecifics during infancy engaged less in grooming others than those predominantly housed with conspecifics during infancy. Astonishingly, all of these former pet and entertainment chimpanzees reacted in a similar way to alterations of group composition as grooming among group members reverted to a more even distribution in stable periods, after a more restricted distribution during unstable periods, throughout the whole 12 years of observation.

We believe that these results might be a valuable addition to the already existing knowledge, especially with respect to care management decisions regarding integration and group formations. For future studies, we suggest using multi-level networks including diverse social behaviours and social proximity as this might give an even better understanding, ideally with a larger sample size and more detailed information on the chimpanzees’ past history.

## Supporting information

S1 Table List of best models, forming part of the subset being used in the averaging process, with vertex strength centrality as dependent variable. VSC models with stability of time period (TPstability), arrival age category (ArrivalAgeCat), sex, predominant housing condition during infancy (PHCinfant) and origin as fixed effects and group composition in a certain time period and ID as random factors in all models. Models are ranked according to the best *AICc*. All models considered here have a *ΔAIC*c lower than 10 compared to the best model (first model listed). Fixed effects included in each model candidate are marked with an X.
(DOCX)

S2 Table List of best models, forming part of the subset being used in the averaging process, with deviation from edge weight disparity as dependent variable. DEWD models with stability of time period (TPstability), arrival age category (ArrivalAgeCat), sex, predominant housing condition during infancy (PHCinfant) and origin as fixed effects and group composition in a certain time period and ID as random factors in all models. Models are ranked according to the best *AICc*. All models considered here have a *ΔAIC*c lower than 10 compared to the best model (first model listed). Fixed effects included in each model candidate are marked with an X.
(DOCX)

S3 Table Results from the ANOVA post hoc test on the VSC Full-model. Signif. codes: ‘***’ ≤0.001 ‘**’ ≤0.01 ‘*’ ≤0.05 ‘.’ ≤0.1 ‘ ’ ≤1.
(DOCX)

S4 Table Results from the ANOVA test on the extended VSC model including the interaction of Origin and Predominant housing condition during infancy (PHCinfant). Results of the Interaction contrast analysis: Results are averaged over the levels of TPstability, ArrivalAgeCat, and Sex with a p-value adjustment based on Tukey method for comparing a family of 4 estimates. Signif. codes: ‘***’ ≤0.001 ‘**’ ≤0.01 ‘*’ ≤0.05 ‘.’ ≤0.1 ‘ ’ ≤1.
(DOCX)

S5 Table Results from the ANOVA post hoc test on the DEWD Full-model. Signif. codes: ‘***’ ≤0.001 ‘**’ ≤0.01 ‘*’ ≤0.05 ‘.’ ≤0.1 ‘ ’ ≤1.
(DOCX)

S6 Table Results from the ANOVA test on the extended DEWD model including the interaction of Origin and Time Period Stability (TPstability). Signif. codes: ‘***’ ≤0.001 ‘**’ ≤0.01 ‘*’ ≤0.05 ‘.’ ≤0.1 ‘ ’ ≤1.
(DOCX)

S1 Fig Graphical representation of the weighted social grooming networks of all 18 Observation Time Periods/Group compositions for both chimpanzee groups (Bilinga B01-B12, Mutamba M01-M06) in chronological order. Nodes represent group members, with green nodes being wild-caught and blue nodes being captive born chimpanzees. Node shape represents the sex, with squares being females and circles being males. Graphs marked with a * are describing stable time periods. The procedures used to create the matrices are described in the method section. Graphs were drawn using the Igraph package in R [92].
(TIF)

S2 Fig Plot of the residual vs. fitted values for vertex strength centrality (VSC). (TIF)

S3 Fig Plot of the residual normality distribution for vertex strength centrality (VSC). (TIF)

S4 Fig Plot of the residual vs. fitted values for deviation from edge weight disparity (DEWD). (TIF)

S5 Fig Plot of the residual normality distribution for deviation from edge weight disparity (DEWD). (TIF)
