# Supplementary material for: Early life experience and alterations of group composition shape the social grooming networks of former pet and entertainment chimpanzees (Pan troglodytes)
Source: PLoS One. 2020 Jan 15;15(1):e0226947. doi: 10.1371/journal.pone.0226947 (PMC6961849; doi:10.1371/journal.pone.0226947)
Supplement: S1 Table — VSC models with stability of time period (TPstability), arrival age category (ArrivalAgeCat), sex, predominant housing condition during infancy (PHCinfant) and origin as fixed effects and group composition in a certain time period and ID as random factors in all models. Models are ranked according to the best AICc. All models considered here have a ΔAICc lower than 10 compared to the best model (first model listed). Fixed effects included in each model candidate are marked with an X. (DOCX) [file pone.0226947.s001.docx]

| **VSC** | **(Int)** | **TP**  **stability** | **Arrival**  **Age Cat** | **Sex** | **PHC**  **infant** | **Origin** | **df** | **logLik** | **AICc** | **delta** | **weight** |
| --- | --- | --- | --- | --- | --- | --- | --- | --- | --- | --- | --- |
| Mod12 | 2.139 |  |  | X | X | X | 7 | -182.688 | 380.4 | 0.00 | 0.452 |
| Mod22 | 2.280 | X |  | X | X | X | 8 | -182.569 | 382.4 | 2.06 | 0.161 |
| Mod9 | 2.274 |  | X | X | X | X | 8 | -182.620 | 382.5 | 2.16 | 0.153 |
| Mod5 | 2.424 | X | X | X | X | X | 9 | -182.495 | 384.6 | 4.26 | 0.054 |
| Mod14 | 2.487 |  |  | X |  | X | 6 | -185.955 | 384.7 | 4.28 | 0.053 |
| Mod30 | 2.689 | X |  | X |  | X | 7 | -185.686 | 386.4 | 6.00 | 0.023 |
| Mod15 | 1.447 |  |  |  | X | X | 6 | -186.858 | 386.5 | 6.08 | 0.022 |
| Mod29 | 2.663 |  | X | X |  | X | 7 | -185.879 | 386.8 | 6.38 | 0.019 |
| Mod16 | 1.834 |  |  |  |  | X | 5 | -188.119 | 386.8 | 6.38 | 0.019 |
| Mod25 | 1.245 |  | X |  | X | X | 7 | -186.713 | 388.4 | 8.05 | 0.008 |
| Mod31 | 2.877 | X | X | X |  | X | 8 | -185.602 | 388.5 | 8.13 | 0.008 |
| Mod21 | 1.553 | X |  |  | X | X | 7 | -186.782 | 388.6 | 8.19 | 0.008 |
| Mod17 | 1.972 | X |  |  |  | X | 6 | -187.959 | 388.7 | 8.28 | 0.007 |
| Mod24 | 1.710 |  | X |  |  | X | 6 | -188.067 | 388.9 | 8.5 | 0.006 |
| Mod23 | 0.5062 |  | X |  | X |  | 6 | -188.567 | 389.9 | 9.5 | 0.004 |
| Mod6 | 0.9414 |  | X |  |  |  | 5 | -189.826 | 390.2 | 9.8 | 0.003 |
