# Supplementary material for: Early life experience and alterations of group composition shape the social grooming networks of former pet and entertainment chimpanzees (Pan troglodytes)
Source: PLoS One. 2020 Jan 15;15(1):e0226947. doi: 10.1371/journal.pone.0226947 (PMC6961849; doi:10.1371/journal.pone.0226947)
Supplement: S2 Table — DEWD models with stability of time period (TPstability), arrival age category (ArrivalAgeCat), sex, predominant housing condition during infancy (PHCinfant) and origin as fixed effects and group composition in a certain time period and ID as random factors in all models. Models are ranked according to the best AICc. All models considered here have a ΔAICc lower than 10 compared to the best model (first model listed). Fixed effects included in each model candidate are marked with an X. (DOCX) [file pone.0226947.s002.docx]

| **DEWD** | **(Int)** | **TP**  **stability** | **Arrival**  **Age Cat** | **Sex** | **PHC**  **infant** | **Origin** | **df** | **logLik** | **AICc** | **delta** | **weight** |
| --- | --- | --- | --- | --- | --- | --- | --- | --- | --- | --- | --- |
| Mod30 | 0.1253 | X |  | X |  | X | 7 | 11.797 | -8.4 | 0.00 | 0.205 |
| Mod17 | 0.2157 | X |  |  |  | X | 6 | 10.425 | -8.0 | 0.44 | 0.164 |
| Mod22 | 0.1016 | X |  | X | X | X | 8 | 12.438 | -7.4 | 1.07 | 0.12 |
| Mod31 | 0.05551 | X | X | X |  | X | 8 | 12.308 | -7.1 | 1.33 | 0.105 |
| Mod21 | 0.2004 | X |  |  | X | X | 7 | 10.769 | -6.4 | 2.06 | 0.073 |
| Mod1 | 0.2656 | X |  |  |  |  | 5 | 8.221 | -5.8 | 2.6 | 0.056 |
| Mod5 | 0.04528 | X | X | X | X | X | 9 | 12.848 | -5.8 | 2.65 | 0.054 |
| Mod27 | 0.2071 | X | X |  |  | X | 7 | 10.439 | -5.7 | 2.72 | 0.053 |
| Mod2 | 0.2993 | X | X |  |  |  | 6 | 8.715 | -4.6 | 3.86 | 0.03 |
| Mod18 | 0.2494 | X |  |  | X |  | 6 | 8.543 | -4.2 | 4.21 | 0.025 |
| Mod26 | 0.196 | X | X |  | X | X | 8 | 10.733 | -4.0 | 4.4 | 0.023 |
| Mod19 | 0.2393 | X |  | X |  |  | 6 | 8.358 | -3.9 | 4.58 | 0.021 |
| Mod28 | 0.2838 | X | X |  | X |  | 7 | 9.109 | -3.1 | 5.38 | 0.014 |
| Mod3 | 0.2757 | X | X | X |  |  | 7 | 8.816 | -2.5 | 5.96 | 0.01 |
| Mod20 | 0.2135 | X |  | X | X |  | 7 | 8.781 | -2.4 | 6.03 | 0.01 |
| Mod14 | 0.2542 |  |  | X |  | X | 6 | 7.472 | -2.1 | 6.35 | 0.009 |
| Mod12 | 0.2208 |  |  | X | X | X | 7 | 8.27 | -1.4 | 7.06 | 0.006 |
| Mod16 | 0.3351 |  |  |  |  | X | 5 | 5.958 | -1.3 | 7.12 | 0.006 |
| Mod4 | 0.2497 | X | X | X | X |  | 8 | 9.31 | -1.1 | 7.32 | 0.005 |
| Mod29 | 0.1878 |  | X | X |  | X | 7 | 7.999 | -0.8 | 7.6 | 0.005 |
| Mod15 | 0.3088 |  |  |  | X | X | 6 | 6.486 | -0.1 | 8.32 | 0.003 |
| Mod9 | 0.1733 |  | X | X | X | X | 8 | 8.621 | 0.3 | 8.7 | 0.003 |
| Mod24 | 0.3319 |  | X |  |  | X | 6 | 5.96 | 0.9 | 9.37 | 0.002 |
