# Supplementary material for: Early life experience and alterations of group composition shape the social grooming networks of former pet and entertainment chimpanzees (Pan troglodytes)
Source: PLoS One. 2020 Jan 15;15(1):e0226947. doi: 10.1371/journal.pone.0226947 (PMC6961849; doi:10.1371/journal.pone.0226947)
Supplement: S3 Table — Signif. codes: ‘***’ ≤0.001 ‘**’ ≤0.01 ‘*’ ≤0.05 ‘.’ ≤0.1 ‘ ’ ≤1. (DOCX) [file pone.0226947.s003.docx]

| **VSC Full-model Post Hoc:**  **Type III Analysis of Variance Table with Satterthwaite's method** | | | | | | |
| --- | --- | --- | --- | --- | --- | --- |
|  | Sum Sq | Mean Sq | Num DF | Den DF | F value | Pr(>F) |
| TPstability | 0.2199 | 0.2199 | 1 | 17.732 | 0.2509 | 0.622637 |
| ArrivalAgeCat | 0.129 | 0.129 | 1 | 12.71 | 0.1472 | 0.707595 |
| Sex | 10.5026 | 10.5026 | 1 | 15.063 | 11.9786 | 0.003473 ** |
| PHCinfant | 7.0448 | 7.0448 | 1 | 15.422 | 8.0348 | 0.01229 * |
| Origin | 13.6113 | 13.6113 | 1 | 11.066 | 15.5242 | 0.002286 ** |
