# Supplementary material for: Early life experience and alterations of group composition shape the social grooming networks of former pet and entertainment chimpanzees (Pan troglodytes)
Source: PLoS One. 2020 Jan 15;15(1):e0226947. doi: 10.1371/journal.pone.0226947 (PMC6961849; doi:10.1371/journal.pone.0226947)
Supplement: S4 Table — Results of the Interaction contrast analysis: Results are averaged over the levels of TPstability, ArrivalAgeCat, and Sex with a p-value adjustment based on Tukey method for comparing a family of 4 estimates. Signif. codes: ‘***’ ≤0.001 ‘**’ ≤0.01 ‘*’ ≤0.05 ‘.’ ≤0.1 ‘ ’ ≤1. (DOCX) [file pone.0226947.s004.docx]

| **VSC Full-model including Interaction between PHCinfant and Origin:**  **Type III Analysis of Variance Table with Satterthwaite's method** | | | | | | |
| --- | --- | --- | --- | --- | --- | --- |
|  | Sum Sq | Mean Sq | Num DF | Den DF | F value | Pr(>F) |
| TPstability | 0.1084 | 0.1084 | 1 | 17.8438 | 0.1224 | 0.7305 |
| ArrivalAgeCat | 0.9008 | 0.9008 | 1 | 10.9216 | 1.0176 | 0.3349 |
| Sex | 12.8005 | 12.8005 | 1 | 13.7881 | 14.4595 | 0.0020 ** |
| PHCinfant | 10.0851 | 10.0851 | 1 | 13.4513 | 11.3922 | 0.0048 ** |
| Origin | 20.8679 | 20.8679 | 1 | 9.2476 | 23.5725 | 0.0008 *** |
| PHCinfant:Origin | 4.3446 | 4.3446 | 1 | 13.2177 | 4.9076 | 0.0449 * |
| **Interaction contrast: Tukey method for comparing the four estimates of PHCinfant: Origin** | | | | | | |
| Contrast | | Estimate | SE | DF | T ratio | P value |
| captive,with - wild,with | | 2.0733 | 0.5316 | 25.21 | 3.900 | 0.0033 ** |
| captive,with - captive,without | | 1.4230 | 0.4652 | 27.20 | 3.059 | 0.0240 * |
| captive,with - wild,without | | 2.3718 | 0.5222 | 24.50 | 4.542 | 0.0007 *** |
| wild,with - captive,without | | -0.6503 | 0.5175 | 24.77 | -1.257 | 0.5980 |
| wild,with - wild,without | | 0.2985 | 0.4487 | 26.49 | 0.665 | 0.9092 |
| captive,without - wild,without | | 0.9488 | 0.5048 | 23.28 | 1.880 | 0.2638 |
