# Supplementary material for: Early life experience and alterations of group composition shape the social grooming networks of former pet and entertainment chimpanzees (Pan troglodytes)
Source: PLoS One. 2020 Jan 15;15(1):e0226947. doi: 10.1371/journal.pone.0226947 (PMC6961849; doi:10.1371/journal.pone.0226947)
Supplement: S5 Table — Signif. codes: ‘***’ ≤0.001 ‘**’ ≤0.01 ‘*’ ≤0.05 ‘.’ ≤0.1 ‘ ’ ≤1. (DOCX) [file pone.0226947.s005.docx]

| **DEWD Full-model Post Hoc:**  **Type III Analysis of Variance Table with Satterthwaite's method** | | | | | | |
| --- | --- | --- | --- | --- | --- | --- |
|  | Sum Sq | Mean Sq | Num DF | Den DF | F value | Pr(>F) |
| TPstability | 0.37143 | 0.37143 | 1 | 15.2589 | 10.154 | 0.006023 ** |
| ArrivalAgeCat | 0.03371 | 0.03371 | 1 | 6.9778 | 0.9215 | 0.369152 |
| Sex | 0.22219 | 0.22219 | 1 | 9.1780 | 6.0741 | 0.035405 * |
| PHCinfant | 0.04208 | 0.04208 | 1 | 8.6841 | 1.1504 | 0.312365 |
| Origin | 0.35146 | 0.35146 | 1 | 6.6089 | 9.6081 | 0.018646 * |
