# Supplementary material for: Early life experience and alterations of group composition shape the social grooming networks of former pet and entertainment chimpanzees (Pan troglodytes)
Source: PLoS One. 2020 Jan 15;15(1):e0226947. doi: 10.1371/journal.pone.0226947 (PMC6961849; doi:10.1371/journal.pone.0226947)
Supplement: S6 Table — Signif. codes: ‘***’ ≤0.001 ‘**’ ≤0.01 ‘*’ ≤0.05 ‘.’ ≤0.1 ‘ ’ ≤1. (DOCX) [file pone.0226947.s006.docx]

| **DEWD Full-model including Interaction between TPstability and Origin:**  **Type III Analysis of Variance Table with Satterthwaite's method** | | | | | | |
| --- | --- | --- | --- | --- | --- | --- |
|  | Sum Sq | Mean Sq | Num DF | Den DF | F value | Pr(>F) |
| ArrivalAgeCat | 0.0297 | 0.0297 | 1 | 7.153 | 0.8154 | 0.3959 |
| Sex | 0.2129 | 0.2129 | 1 | 9.255 | 5.8445 | 0.0381 * |
| PHCinfant | 0.0437 | 0.0437 | 1 | 8.765 | 1.1986 | 0.3028 |
| TPstability | 0.3723 | 0.3723 | 1 | 16.221 | 10.2215 | 0.0055 ** |
| Origin | 0.2941 | 0.2941 | 1 | 8.058 | 8.0752 | 0.0216 * |
| TPstability:Origin | 0.0053 | 0.0053 | 1 | 91.883 | 0.1463 | 0.7030 |
