# Supplementary figures and images for: Early life experience and alterations of group composition shape the social grooming networks of former pet and entertainment chimpanzees (Pan troglodytes)
Source: PLoS One. 2020 Jan 15;15(1):e0226947. doi: 10.1371/journal.pone.0226947 (PMC6961849; doi:10.1371/journal.pone.0226947)

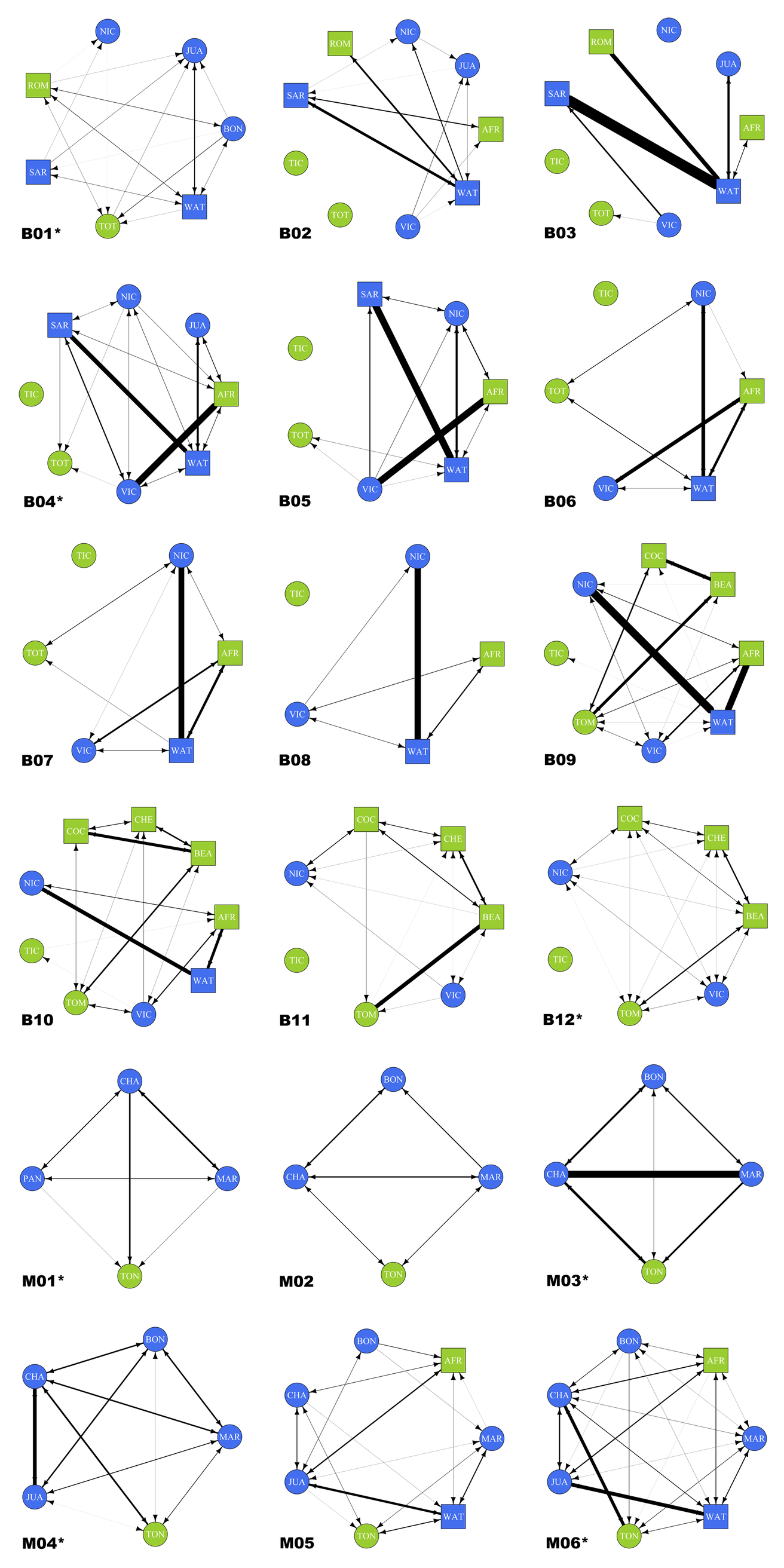

Supplement: S1 Fig — Nodes represent group members, with green nodes being wild-caught and blue nodes being captive born chimpanzees. Node shape represents the sex, with squares being females and circles being males. Graphs marked with a * are describing stable time periods. The procedures used to create the matrices are described in the method section. Graphs were drawn using the Igraph package in R [92]. (TIF) [file pone.0226947.s007.tif]

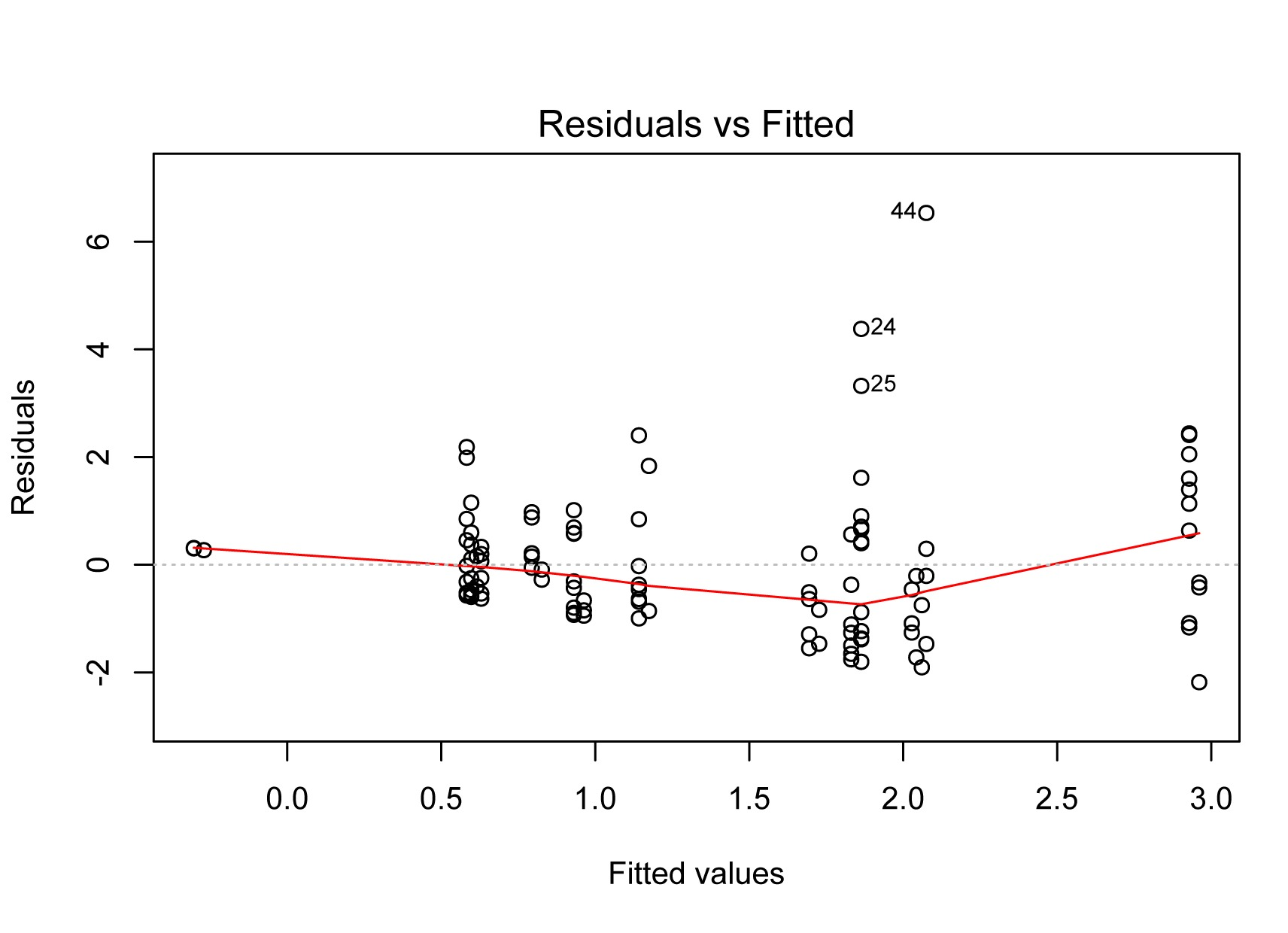

Supplement: S2 Fig — (TIF) [file pone.0226947.s008.tif]

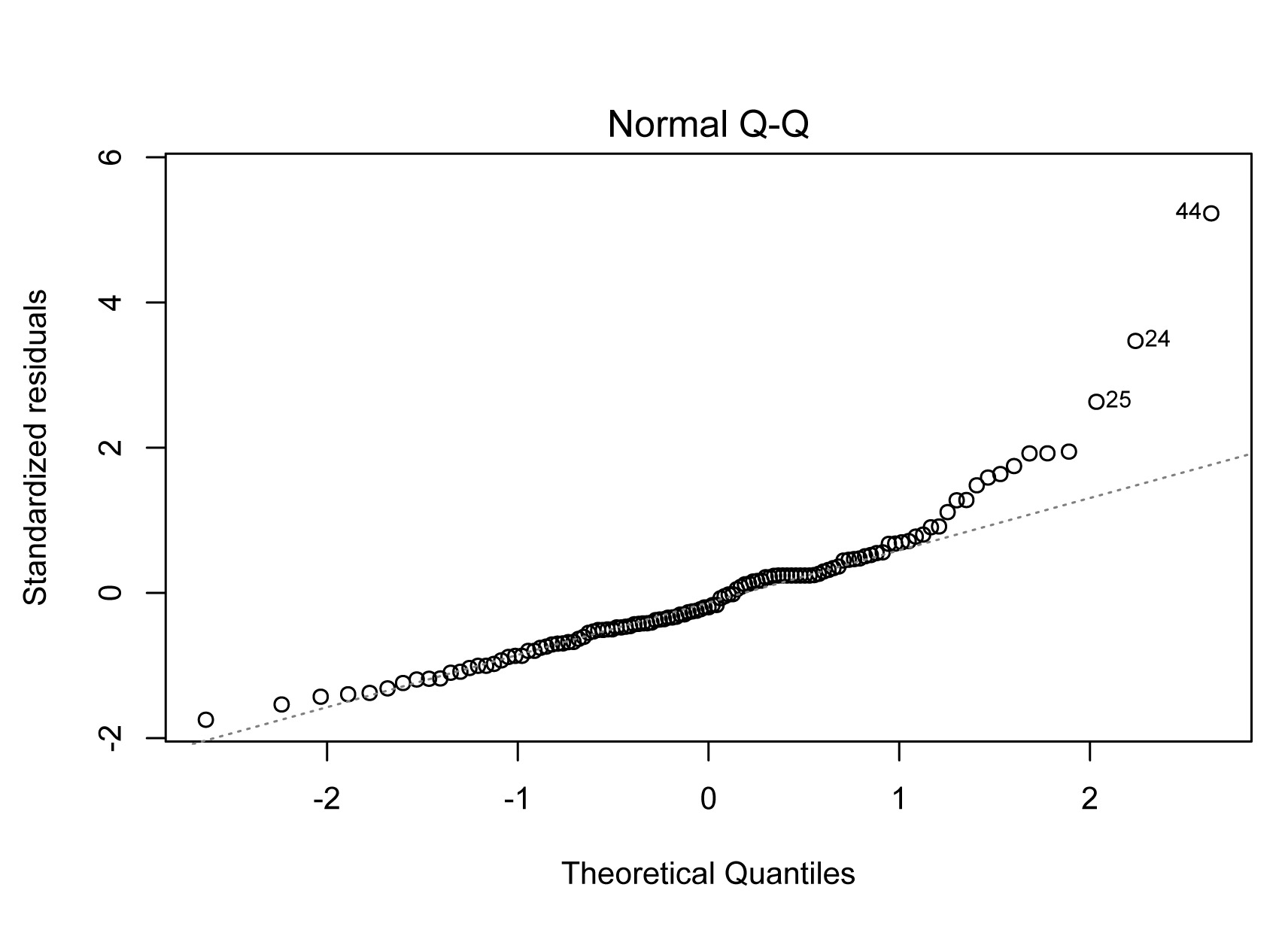

Supplement: S3 Fig — (TIF) [file pone.0226947.s009.tif]

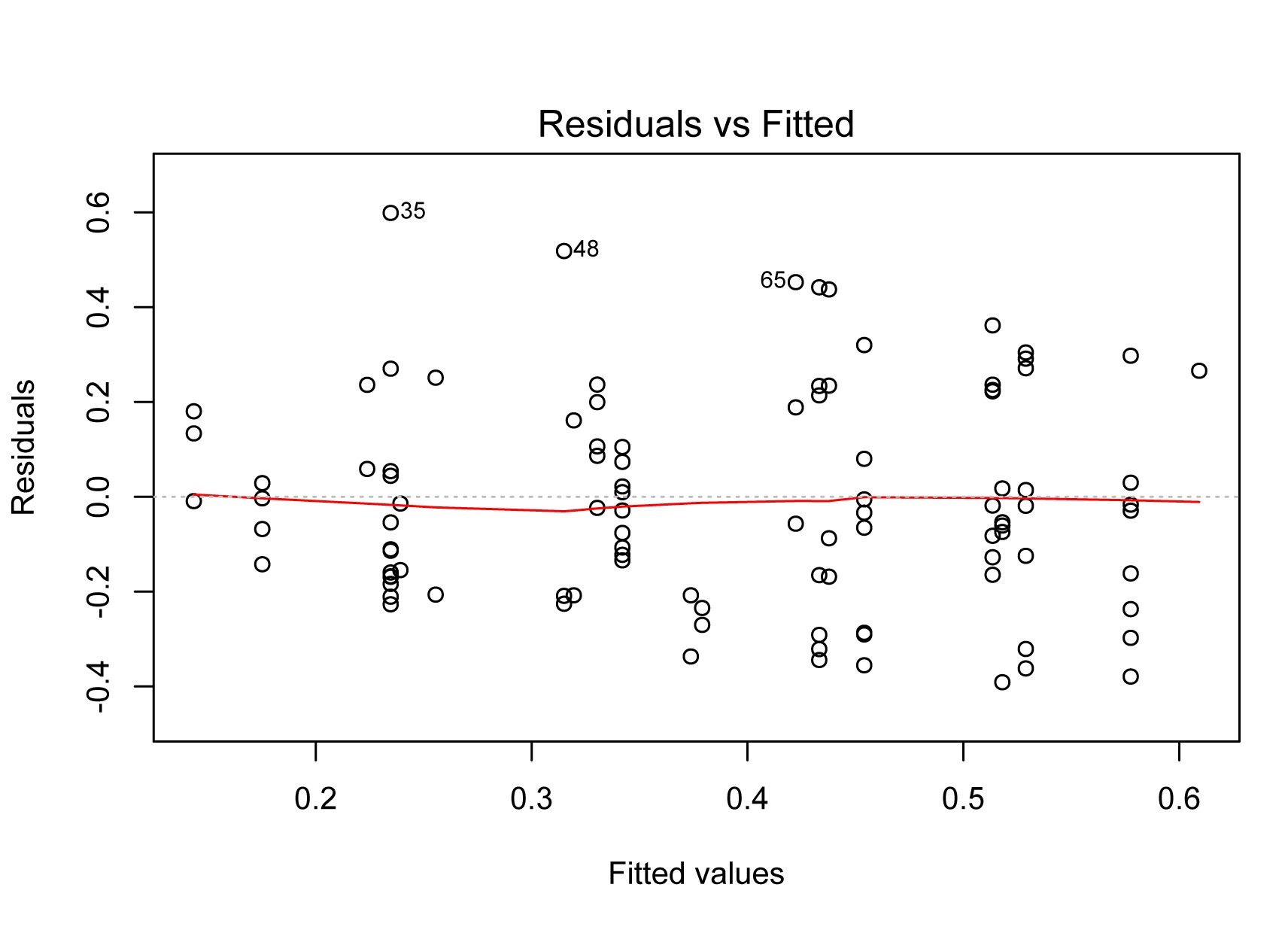

Supplement: S4 Fig — (TIF) [file pone.0226947.s010.tif]

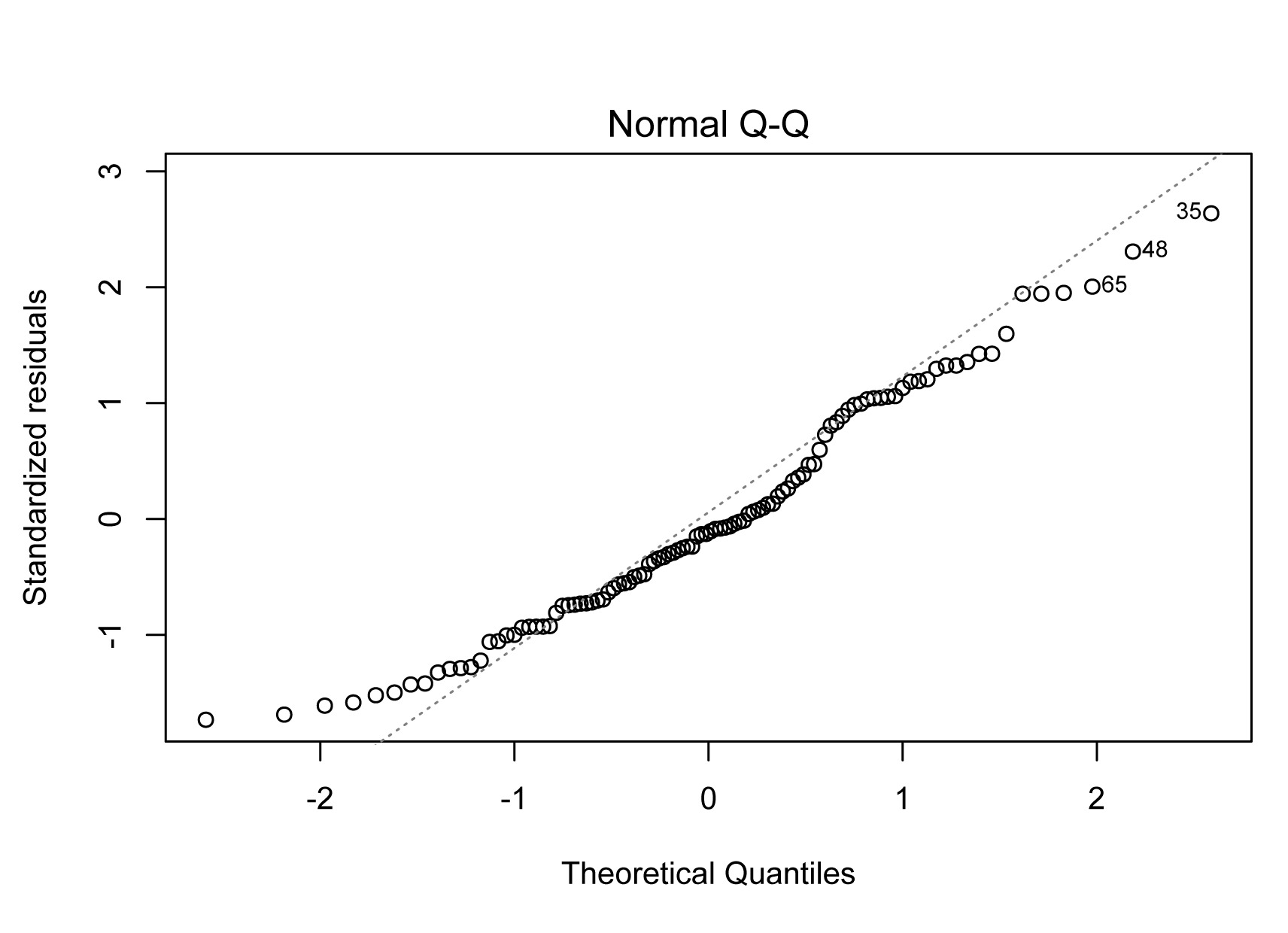

Supplement: S5 Fig — (TIF) [file pone.0226947.s011.tif]
